# Experimental characterization of complex atmospheric flows: A wind turbine wake case study

**DOI:** 10.1126/sciadv.adw8524

**Published:** 2025-11-21

**Authors:** Nikolas Angelou, Mikael Sjöholm, Torben Krogh Mikkelsen

**Affiliations:** Department of Wind and Energy Systems, Technical University of Denmark, Frederiksborgvej 399, 4000 Roskilde, Denmark.

## Abstract

Our current understanding of the interaction between the atmosphere and surface obstacles crucial for boundary-layer meteorology, forestry, urban climate, wind engineering, and wind energy is limited mainly to observations acquired in wind tunnel experiments and flow predictions from computational fluid dynamic models. Here, as a case study, we present spatially distributed measurements of a utility-scale wind turbine’s wake using three wind lidars that synchronously scan a volume of the atmosphere. The results reveal not only information of the mean wake flow generated by a wind turbine, such as the distribution of the velocity deficit and its spatial gradients, but also observations of the momentum fluxes that control the interaction between the wake and the surrounding atmospheric flow, which is essential for optimizing wind energy production. The presented remote sensing methodology represents a paradigm shift for atmospheric field studies, enabling unprecedented flow observations.

## Teaser

Synchronized scanning Doppler lidars enable experimental characterization of wind and turbulence in complex atmospheric flows.

## INTRODUCTION

Our knowledge of the physics that governs complex flows in the atmospheric boundary layer is a limiting factor for the future development of wind turbines and their efficient operation in wind farms (*1*, *2*), which are cornerstones for an effective green transition in the global energy production. A particularly complex and crucial flow in wind energy research is the wake generated by the operation of wind turbines (*3*). A wake in the vicinity of a wind turbine is a rotating and turbulent flow with a velocity deficit that propagates downwind from the turbine in the wind direction. Furthermore, as wind flows through an operating wind turbine rotor, the ambient atmospheric turbulence is mixed with turbulence generated by the interaction of the wind and the rotating blades. This process has an impact on both the wake characteristics and their downwind propagation. The accurate prediction of the mean velocity deficit and of the added turbulence is paramount from a wind energy aerodynamics perspective since the geometry, the dimensions, the design, and the operational parameters of wind turbines determine the outcome of their interaction with various inflow conditions. These parameters thus drive not only the power production but also the fatigue and potential damage loads of wind turbines, as well as the degree of flow distortion caused by the presence of the turbines. The latter has an impact on the adjacent wind turbines and thus determines the productivity and the life expectancy of a whole wind farm, which are vital parameters for a viable economic wind farm planning. On a larger perspective, these flow details are also crucial for assessing the impact of wind turbines (*4*, *5*) and wind farms (*6*) on the local environment.

Wake flows generated by the operation of wind turbines have mainly been examined in controlled simplified experiments either in wind tunnel facilities or using Reynolds-averaged Navier-Stokes and large eddy simulation computational fluid dynamic models that have enhanced our understanding of the interaction between wind and wind turbines (*3*, *7*). An advantage of numerical simulations of wakes is that the model output provides flow predictions over crosswind rotor planes at many different locations. Spatially distributed measurements of the wind vector can be achieved in wind tunnels through optical techniques (i.e., particle image velocimetry measuring technique) as presented, for example, in (*8*–*10*) or by moving an in situ anemometer in different locations, e.g., (*11*, *12*). This way, it is feasible to study the spatiotemporal characteristics that describe the flow distortion in the wake of a wind turbine. Yet, the main challenge of these studies is the generation of realistic atmospheric wind conditions that wind turbines interact with.

However, the study of utility-scale wind turbine wakes that propagate in the atmosphere during natural environmental conditions has been limited until recently. The primary reason is that, traditionally, atmospheric field experiments have been conducted using meteorological masts based on in situ observations of the wind conditions. Although in situ observations are characterized by a high temporal resolution, the acquisition of distributed measurements over the large volumes necessary for the study of wakes still remains challenging. An opportunity to meet this challenge arose with the development of the optical remote sensing technique of light detection and ranging (lidar), which provided a new modality in the study of wakes under real atmospheric conditions. The first studies of wind turbine wakes were conducted using nacelle-mounted scanning wind lidars with a focus on the near-wake region (*13*–*15*). The near-wake region corresponds to the area where both the physical characteristics (i.e., shape and dimensions) of the wind turbine and the rotating blades of the rotor affect the flow (*16*). The development of the robustness of scanning wind lidars also enabled the characterization of far wakes using either ground-based (*9*, *17*–*19*) or nacelle-mounted (*20*–*23*) wind lidars along with sensors installed on meteorological masts used in a complementary manner. The former provided a visual depiction of the mean spatial characteristics of the flow and the latter time series of the wind in specific locations that could be used to study the temporal characteristics. The advantage of nacelle-mounted lidars is that they can follow the flow direction on the lee side while pointing downwind. The ground-based configurations on the contrary only enable the interpretation of lidar measurements for a wake characterization during specific wind directions, e.g., (*24*). This was partially tackled using dual lidar configurations (*25*), in which two Doppler lidars were used in a synchronous manner to monitor the wake flow. The use of the aforementioned wind lidar configurations assumes that the mean wind flow aligns either with a specific wind direction or with a horizontal plane (and thus the mean vertical wind component is negligible). These assumptions pose a limitation on studying complex flows since wind is a three-dimensional (3D) vector. Thus, three independent measurements are required over the same air volume to fully characterize the wind vector as reported in (*26*, *27*).

In this study, we demonstrate that a wake characterization can be realized using a remote sensing system that consists of three Doppler wind lidars that synchronously scan a user-defined volume of air. For this purpose, we use the WindScanner research infrastructure, developed at the Wind and Energy Systems Department at the Technical University of Denmark (DTU) (*28*). The WindScanner has been used in measurement campaigns to study the mean 3D wind inflow and the wake of wind turbines over homogeneous (*29*, *30*) and complex (*31*–*33*) terrains, as well as the wake behind windbreaks (*34*) and solitary trees (*35*). In addition to the mean wake flow, the WindScanner has been used to study the turbulence intensity of a wind turbine wake along vertical and horizontal profiles (*36*). Here, we demonstrate that using the WindScanner, it is possible to extract not only information of the mean flow, such as the distribution of the wake deficit and the gradients of the wind, but also distributed observations of the momentum fluxes. The distribution and the magnitude of the momentum fluxes define the interaction of the flow within the wake with the free flow. This is important since these flow characteristics determine the propagation of the wake and thus affect the operation of wind farms. So far, this type of measurements has been possible only in wind tunnel studies where the wake flow behind small-scale wind turbines has been studied under idealized inflow conditions. However, utility-scale wind turbines operate in the planetary boundary layer, where the characteristics of the terrain and the stratification of atmospheric temperature have a notable impact on the wind conditions. Thus, multiple scanning wind lidars that can probe a volume of air in a synchronized manner facilitate advanced studies of complex flows that pave a path toward a better understanding of the atmospheric physics that govern the wind flow.

### Field campaign

**Fig. 1.**
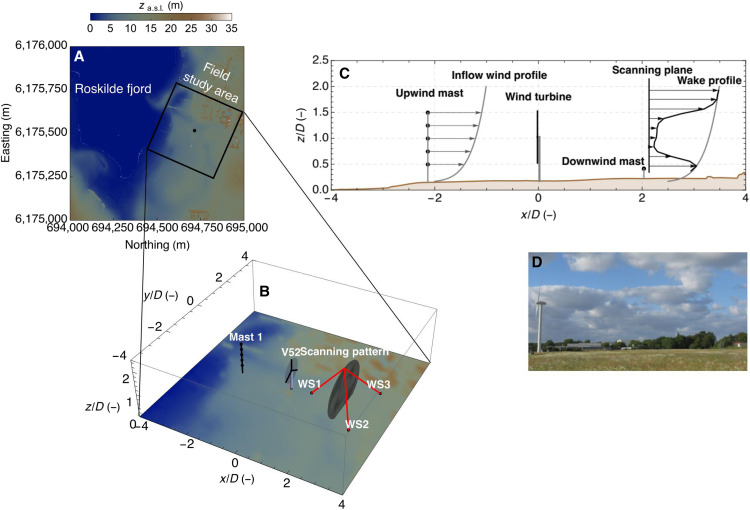
Field campaign setup. (**A**) Elevation map of the eastern coast of the Roskilde fjord and (**B**) perspective view of the field study area, where (i) the wind turbine, (ii) the 75-m tall upwind mast used to measure the inflow conditions relative to the wind turbine, and (iii) the wind lidars (WS1, WS2, and WS3) used to measure the wake over a scanning plane are depicted. a.s.l., above sea level. (**C**) A side view of the measuring setup where in addition to the aforementioned upwind mast, a 10-m tall mast installed downwind from the wind turbine is shown and (**D**) a photograph of the V52 wind turbine and of the downwind terrain over which the wake flow is propagating. The *x* axis of the coordinate system presented in (B) and (C) points toward 110° relative to the north.

**Fig. 2.**
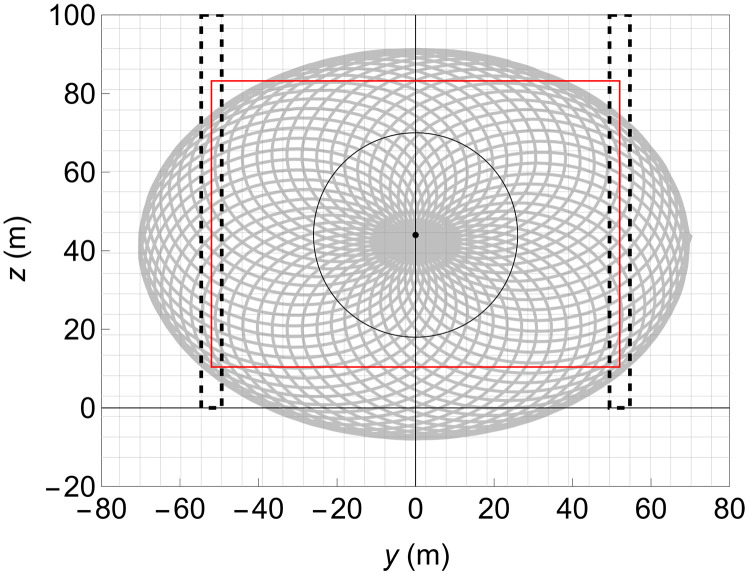
Scanning pattern. The scanning pattern performed by the three wind lidars (shown in gray) and the periphery of the rotor (indicated by a black circle) when the wind turbine yaw aligns to the coordinate system used (i.e., yaw direction of 290°). The two rectangles marked by dashed black lines highlight the areas used to estimate the free wind profiles, and the red square is the domain where we investigated the wake properties.

This section briefly describes the field campaign that was conducted to achieve a wake characterization using three scanning wind lidars. Detailed information regarding the field campaign, the dataset acquired, and the data postprocessing is presented in Materials and Methods. The three wind lidars of the WindScanner research infrastructure were installed in a triangular configuration downwind of a utility-scale V52 wind turbine, with a rotor diameter *D* = 52 m. The wind turbine is located at the Risø Campus of DTU (see Fig. 1, A and B, where the position of each of the three wind lidars, denoted as WS1, WS2, and WS3, is presented). When the wind blows from the sector between west and west-northwest, it travels over an upwind fetch of ~6 km of water before reaching the wind turbine. Furthermore, for that particular wind direction, the terrain downwind of the wind turbine is homogeneous and relatively flat for a distance of almost four rotor diameters (Fig. 1, C and D). Therefore, the topological features of the terrain are expected to have a negligible impact on the wake propagation. The wind lidars were configured to scan over a vertical plane that was situated at a downwind distance of 110 m (corresponding to 2.1 *D*) from the wind turbine (see Figs. 1, C and D, and 2). At this distance, the wake flow is measured within the near-wake region, which typically extends up to four rotor diameters downwind. The three wind lidars were scanning, in a continuous mode along an elliptical rosette pattern with minor and major axes equal to 100 m (1.9 *D*) and 140 m (2.7 *D*), respectively (see Fig. 2). The scanning pattern aimed to cover the whole-wake cross section, even if that was translated laterally because of variations in the wind direction. One scan was completed within 60 s, and the sampling rate was equal to ~322 Hz to maximize the spatial resolution per scan. The trajectories of the three wind lidars were programmed and controlled by the same computer, and, thus, the measurements were synchronized in time and colocated in space. The acquired data were gathered in a vertical grid that was defined in a right-handed Cartesian coordinate system, whose *x* axis was aligned to the mean wind direction. The grid consisted of square-shaped cells with a dimension of 5.2 m by 5.2 m, corresponding to 0.1 *D*.

## RESULTS

### Inflow conditions

An assessment of the accuracy of the wind characteristics obtained by the WindScanner is performed through the comparison of the vertical profiles of the free wind, i.e., undisturbed by the operation of the wind turbine, with those measured by the in situ sensors installed at the upwind meteorological mast. For this purpose, we select wind lidar measurements from two vertical regions in the scanning plane determined by −1.05<y/D<−0.95 and 0.95<y/D<1.05 , respectively (the location of which are depicted in Fig. 2). In these regions, we estimate the first-order (mean) and second-order (variances and covariances) moments of the wind vector at each measurement point. For the description of the 3D wind vector U={u,v,w} , where *u*, *v*, and *w* denote the longitudinal, transverse, and vertical velocity components relative to the mean wind direction, we use the Reynolds decomposition to separate the notation of the mean (denoted by an overline) and the fluctuating part (denoted by ′) of each component. We select an ensemble of 22 measurement periods for the present case study. Each ensemble period has a duration of 30 min, and it is characterized by the same features: (i) wind speed at hub height (i.e., 7 m s^−1^), (ii) alignment between the mean wind and wind turbine yaw direction (i.e., ±5°), and (iii) atmospheric stability (more information about the selection criteria and the postprocessing of the data can be found in Materials and Methods). The wind vector statistics presented correspond to the ensemble mean values (denoted by <>).

**Fig. 3.**
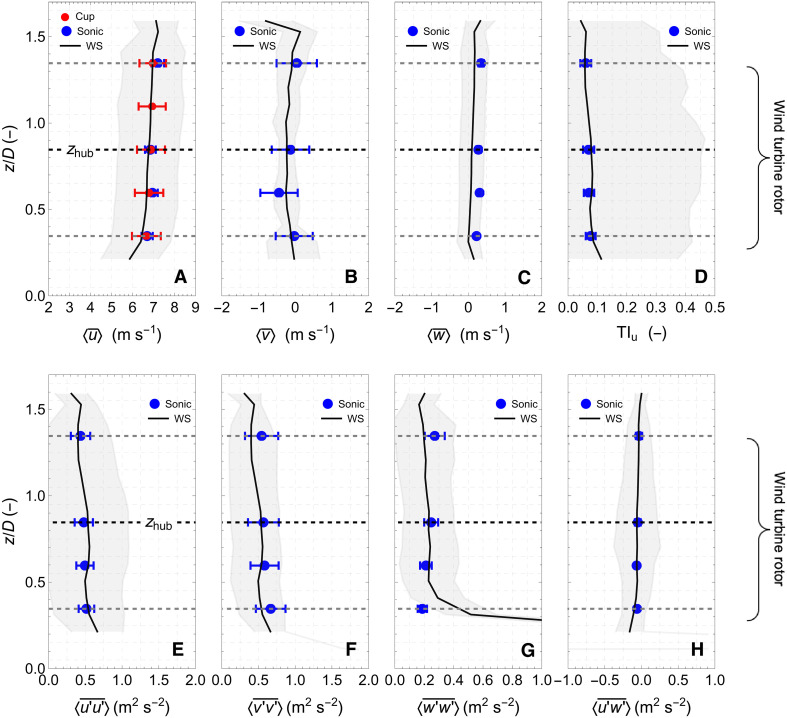
Vertical profiles of the inflow wind vector. (**A** to **C**) Vertical profiles of the mean components of the 3D vector, (**D**) of the turbulence intensity of the longitudinal component of the wind, (**E** to **G**) of the measured covariances ( $<\;\bar{u^{\prime }u^{\prime }}\;>$ , $<\;\bar{v^{\prime }v^{\prime }}\;>$ , and $<\;\bar{w^{\prime }w^{\prime }}\;>$ ), and (**H**) of the vertical momentum flux $<\;\bar{u^{\prime }w^{\prime }}\;>$ as a function of the height normalized by the rotor diameter. The gray area and the error bars depict one SD about each mean. The heights of the bottom and the top blade tips, as well as of the hub ( $z_{\text{hub}}$ ), are depicted by black dashed lines.

The mean wind speed profiles measured by the WindScanner in the area outside the wake region are compared with the sonic and cup anemometer measurements, which are depicted with blue and red markers, respectively, in Fig. 3 (A to C). We observe that the mean values based on the wind lidars are, within the uncertainty level presented by error bars, consistent with the estimated values from the in situ sensors, providing insight into the wind shear and veer. The longitudinal wind speed increases between the bottom and the top wind turbine blade tips by 0.6 m s^−1^ corresponding to a weak vertical wind shear of ~0.01 s^−1^. Regarding the profile of the mean transverse component, the sonic anemometers report an increased speed at a height of 0.6 z/D . Above that height the transverse wind speed component increases from −0.44 to 0.05 m s^−1^, indicative of a slight wind veer of ~0° to 1°/m. We find the most notable difference between WindScanner and the sonic anemometers in the vertical mean wind component. Here, we observe higher values measured by the sonic anemometer at all heights. The mean difference averaged over the four heights is equal to 0.21±0.02 m s^−1^. This difference is attributed to the terrain induction of the flow. At the location of the meteorological mast, the terrain has a slope of 4°, which will induce a small vertical component on the flow when wind originates from the shore (*37*). In contrast, the terrain is relatively flat at the location of the lidar-measuring plane (see Fig. 1C). The slight increase in the vertical component at the lower heights z/D<0.2 is caused by erroneous estimations of the vertical component of the wind vector by the wind lidars due to the low elevation angle of the line of sight (for a discussion about this limitation, see Materials and Methods).

In addition to the mean values of the free wind vector, we also compare the corresponding variances and covariances estimated from the mast (sonic anemometer) and WindScanner measurements. The turbulent fluctuations of the wind vector based on the measurements of the three wind lidars are going to be biased because of their spatial averaging volume (*26*, *27*, *38*). However, we find that the turbulence intensity (TI) of the downwind longitudinal wind component measurements in Fig. 3D are nearly at the same level for both the sonic and wind lidar measurements. This almost too good agreement between the wind lidar and the sonic anemometer measurements could be attributed to the development of an internal boundary layer due to the interface between the sea and the land that could result in an increased turbulence between the position where the scanning plane was located and the position of the upwind mast, compensating, to some extent, for the filtering that is induced by the wind lidar spatial averaging. This could explain why we find in Fig. 3E that the variances of the longitudinal wind component from the wind lidar are comparable but 7 to 10% larger than the corresponding sonic anemometer values at the height range between 18 and 44 m. This trend is not observed in neither the transverse component in Fig. 3F nor the vertical component in Fig. 3G of the wind vector. Furthermore, in the vertical variance, we observe a slight increase in the values estimated using the sonic anemometer data for heights between 0.6 and 1.3 *D*, while the variance in the WindScanner measurements remain approximately constant. Last, in Fig. 3H, we report values of the vertical momentum flux $<\;\bar{u^{\prime }w^{\prime }}\;>$ and observe a generally good agreement between the vertical profiles measured by the wind lidars and the sonic anemometers, where the momentum flux tends to lower values with height, as anticipated in a turbulent boundary layer. Overall, the inflow conditions encountered by the wind turbine can be considered to be characterized by a homogeneous wind field with low wind shear and veer and moderate turbulence levels, since, at the hub height, both the sonic anemometers and the WindScanner measure equal turbulence intensities of 0.07 and the difference of turbulence intensities between the bottom and top blade tip is as small as 0.01.

### Wake flow characteristics

**Fig. 4.**
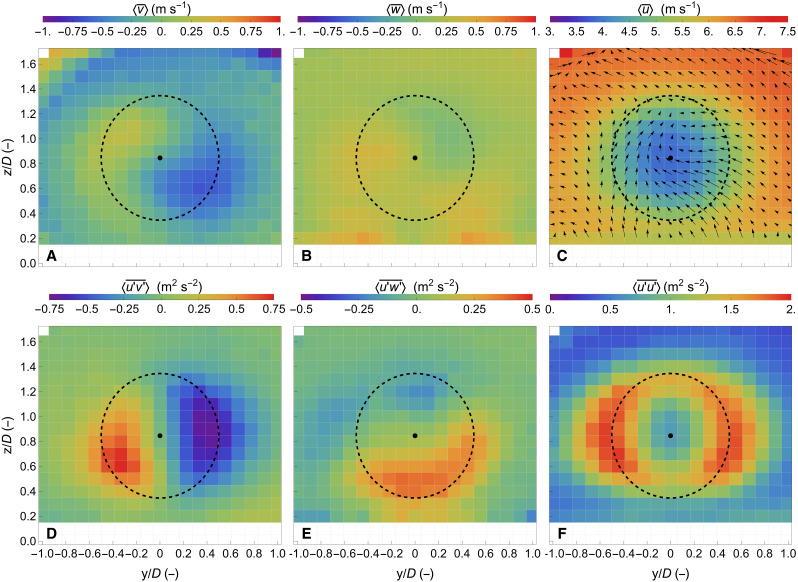
Cross section of the wake flow. The mean [(**A**) transverse, (**B**) vertical, and (**C**) longitudinal] and second-order moments [(**D**) $<\;\bar{u^{\prime }v^{\prime }}\;>$ , (**E**) $<\;\bar{u^{\prime }w^{\prime }}\;>$ , and (**F**) $<\;\bar{u^{\prime }u^{\prime }}\;>$ ] of the wind vector components in the wake of a wind turbine. The dashed circle marks the rotor diameter of 52 m, centered at the hub height of 44 m. The streamlines of the crosswind vector $\left\{\;<\;\bar{v}\;>,<\;\bar{w}\;>\;\right\}$ in the wake are depicted with arrows in (C).

In Fig. 4, we present the ensemble average of the three components of the wind vector in a 2D vertical rectangular plane. The measurements in this plane contain information about both the free wind along with the cross section of the wake flow. In Fig. 4A, we observe that the transverse wind component $<\;\bar{v}\;>$ over a slightly larger area than the one of the wind turbine rotor has increased values in comparison with the free flow and, in addition, a different sign between the left (i.e., y/D<0 ) and right (i.e., y/D>0 ) sides, indicating a rotation of the flow inside the wake. Increased vertical wind component values $<\;\bar{w}\;>$ are also found in the region outside the rotor-swept area in Fig. 4B, in agreement with the flow trends of the transverse component. However, the magnitude of the vertical wind component variations is slightly lower than the ones observed in the $<\;\bar{v}\;>$ component. In this figure, we also find that a slight positive vertical wind component characterizes the free flow, as shown in Fig. 3C, and, furthermore, an increase in the estimated vertical component values at the height of z/D=0.2 . The latter is attributed to the limitation of the configuration used for accurately measuring the vertical wind component when the line-of-sight directions of the three wind lidars are almost coplanar close to the ground.

When combining the transverse and the vertical wind components, we reconstruct the crosswind vector $\left\{\;<\;\bar{v}\;>,<\;\bar{w}\;>\;\right\}$ , namely, the motion of the flow over the cross-sectional plane, depicted by vector arrows in Fig. 4C. We observe that the flow is moving upward at the left side of the wake (i.e., y/D<0 ), a direction consistent with the expected rotation of the wake, which is in the opposite direction of the rotation of the wind turbine blades. On the right side (i.e., y/D>0 ), a downward motion is not visible, as expected. We hypothesize that this is due to the slight transverse wind component of the free flow. Figure 4C also illustrates the velocity deficit in the wake of the flow. A velocity deficit with a maximum reduction of 40% characterizes the wind flow in an area that reaches 1.5 times the rotor radius, and it is found to be symmetric around the rotation axis. Despite the close distance between the measured plane and the wind turbine (i.e., x/D=2.1 ), the velocity deficit is characterized by a profile that can be approximated by a Gaussian distribution, particularly in the horizontal direction (see Wake center section).

Figure 4 also presents the second-order moments in the wake flow, particularly the horizontal $<\;\bar{u^{\prime }v^{\prime }}\;>$ (Fig. 4D) and vertical $<\;\bar{u^{\prime }w^{\prime }}\;>$ (Fig. 4E) momentum fluxes, as well as the variance (Fig. 4F) of the longitudinal wind component. We observe that the horizontal fluxes $<\;\bar{u^{\prime }v^{\prime }}\;>$ are increased in the sides of the wake, with positive values on the left side and negative values on the right side. The sign of the fluxes shows that momentum transfers from the periphery to the center of the wake in a mechanism that promotes the recovery of the flow. The magnitude of the fluxes is similar at both sides with slightly larger values at the right side of the flow. Furthermore, the area of the increased momentum flux values at the right side is more prominent than at the left side, indicating that the fluctuations of the longitudinal and transverse components are more correlated on the right side than on the left side. Again, this could be attributed to the transverse component of the free flow. Last, we notice that the distribution of the increased values of the momentum flux is not symmetric with respect to the vertical axis, but, instead, it is rotated by an angle of ~20°.

In terms of the vertical momentum flux, positive values at the bottom and negative values at the top are detected, as expected. However, again, we can highlight two interesting features: First, the vertical flux values are higher at the bottom than at the top part of the wake. Similar behavior of the flow in the near wake region has been reported in the work of Wu and Porté-Agel (*39*) using a high-fidelity computational fluid dynamic model to investigate the impact of the wind shear on the wake characteristics over the wake cross section at different downwind distances. They report that there could be higher values at the bottom than at the top of the wake in the case of low wind shear, which is typical of offshore wind conditions. Second, consistent with the results presented in Fig. 4D, we see that the distribution of the vertical fluxes is not symmetric along the transverse *y* axis but is instead rotated by an angle of 16°. In the work of Wu and Porté-Agel (*39*), the bottom tip of the wind turbine was located at z/D=0.38 , similar to the distance of the bottom blade tip from the ground z/D=0.33 in the present study. This rotation is reported also in the work of Chamorro and Porté-Agel (*11*) at x/D=5 ; however, the rotation of the axis has an opposite sign than in the present study.

Furthermore we present the variance of the longitudinal wind component in Fig. 4F, where we observe increased values along the whole periphery of the wake. The values are larger at the sides of the wake than in the top and bottom parts. This phenomenon could be attributed to how the ensemble average is estimated in a fixed frame since only the variations in the transverse direction are taken into account (the translation of the wake center in the vertical was found to be within a grid cell; more information regarding the wake center tracing is presented in Materials and Methods). However, in general, as reported in (*39*), the variance of the longitudinal wind component at the top of the wake increases with the wind shear.

### Wind speed gradients in the wake

**Fig. 5.**
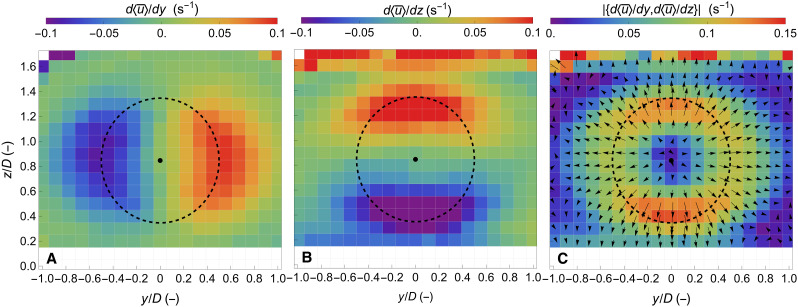
Cross sections of the spatial gradient of the longitudinal wind component. (**A** and **B**) The transverse and vertical gradients of the longitudinal wind component (denoted $d<\;\bar{u}\;>\;/\;\mathrm{dy}$ and $d<\;\bar{u}\;>\;/\;\mathrm{dz}$ , respectively). (**C**) The norm and the direction of the two longitudinal gradients shown in (A) and (B). The dashed circle marks the rotor diameter of 52 m, centered at the hub height of 44 m.

An essential feature of complex atmospheric flows is the spatial gradient of the wind speed along the main wind direction. Specifically, the spatial rate of change of the longitudinal wind component, along the transverse and vertical directions, signifies the momentum exchange between different atmospheric flow layers. This is particularly relevant for the study and the modeling of wind turbine wakes since the dissipation of the wake due to its mixing with the free flow takes place in those areas, where an increase in the spatial gradient is observed (*40*). In Fig. 5 (A and B), we present the transverse $d<\;\bar{u}\;>\;/\;\mathrm{dy}$ and the vertical $d<\;\bar{u}\;>\;/\;\mathrm{dz}$ gradients of the longitudinal wind speed component. In the case of the $d<\;\bar{u}\;>\;/\;\mathrm{dy}$ , an increase in the gradients at the sides of the wake is found, with slightly higher values at the left side of the wake, as seen from the lee side of the wind turbine. A similar increase is observed in the vertical gradient $d<\;\bar{u}\;>\;/\;\mathrm{dz}$ but, instead, on the top and bottom sides of the wake, as can be seen in Fig. 5B. These features highlight that momentum is transferred from the free flow toward the center of the wake from all radial directions with respect to the center of the rotor, as shown in Fig. 5C, where the norm of the two gradients is presented along with its direction. We can see that the direction of the two gradients (depicted using black arrows) is pointing outside from the center of the wake corresponding to a momentum transfer in the opposite direction (*41*).

## DISCUSSION

The biggest challenge in using three Doppler lidars for studying atmospheric turbulence is the inherited measuring characteristic that Doppler wind lidars rather than point measurements provide observations averaged within a probe volume. This poses a limitation to the detection of small wind fluctuations, which leads to an underestimation of the measured atmospheric turbulence in comparison to in situ sensors that are typically installed on meteorological masts (i.e., cup and sonic anemometers) (*38*). The degree of underestimation is determined by the size of the probe volume, which, in the case of a continuous-wave (cw) Doppler lidar, as the ones used in this study, is defined by both the optical characteristics of the lidar (i.e., telescope aperture radius and laser wavelength) and the measuring distance (*42*). The smaller the scales of the atmospheric turbulence with respect to the probe volume of the lidar, the stronger the attenuation of the measured turbulence fluctuation by wind lidar will be, an effect that can be expressed as a low-pass filter (*43*). Although efforts to minimize the probe volume have been attempted (*44*), this still poses a research challenge for the remote sensing scientific community. The effect of the probe length of the three wind lidars used in this study is presented in Fig. 3 (D to G) and in the “Wind vector estimation” section.

The measuring setup used enabled the measurement of the wake within distances between ~63 and 133 m downwind of the wind turbine, corresponding to (1.2 *D* to 2.6 *D*). The downstream distance where the wake characteristics were studied was selected on the basis of the operational specifications of the wind lidars and the practical limitations regarding the easy installation of the wind lidars. Furthermore, an additional criterion was to have the instruments as close as possible to the measuring area to ensure limited wind lidar probe volumes. If longer measuring distances were required, then the wind lidars should be placed either farther apart or at a farther downwind distance.

The investigation of the wake characteristics was based on the terrain homogeneity of the field study, which simplified the application of our measuring methodology. A terrain heterogeneity at the upstream direction would make it difficult to characterize the inflow conditions using only one upwind meteorological mast. In that case, another set of 3D scanning Doppler lidars would be required to map the inflow over the area of interest. Furthermore, terrain heterogeneity at the downwind direction would introduce spatial variations in the flow in the wake. In this case, the impact of the spatial variation of the flow due to terrain heterogeneity could be alleviated by measuring the flow variation when the wind turbine was not operating.

The presented measurement methodology enables the acquisition of spatially distributed measurements of the 3D wind vector under natural atmospheric conditions. This is a turning point in the study of complex atmospheric flows since this type of measurement could, until now, only be acquired in wind tunnel experiments. The acquired data are useful to not only provide an estimation of the first order moments (mean value) of the three components of the wind vector but also provide insight into the distribution of the second order moments (variance and covariance) of the turbulent fluctuations. Thus, it is possible to identify regions with high gradients and high momentum transfer, which is particularly relevant for the study of complex flows such as those encountered in wakes generated by wind turbines. This feature is particularly relevant for the validation of parameterizations that are implemented in computational fluid dynamic and engineering models to study and simulate atmospheric flows. Thus, a measuring methodology based on scanning wind lidars that synchronously acquire colocated observations can pave the way for new discoveries regarding the physics that govern the interaction of wind with surface obstacles in the fields of boundary-layer meteorology, forestry, urban climate, wind engineering, and wind energy.

## MATERIALS AND METHODS

### Field campaign

The study we present was performed using data acquired between 2 and 13 July 2019 during a field campaign that took place at the Risø Campus of the DTU. The Risø Campus is located on the eastern shore of the Roskilde fjord. A V52 wind turbine (Vestas Wind Systems A/S), with a hub height of 44 m, a rotor diameter of 52 m, and a rated power of 850 kW at a hub height wind speed of 17 m s^−1^, was located 150 m east of the coast. The field campaign objective was to characterize the wind conditions at the lee side of the wind turbine when the wind was flowing from the fjord (i.e., west-northwest direction), which is a typical wind direction for the area of the study. For this purpose, three WindScanner wind lidars developed by DTU were deployed in a triangular configuration at the lee side of the wind turbine to measure the wake conditions.

### Short-range WindScanner

**Fig. 6.**
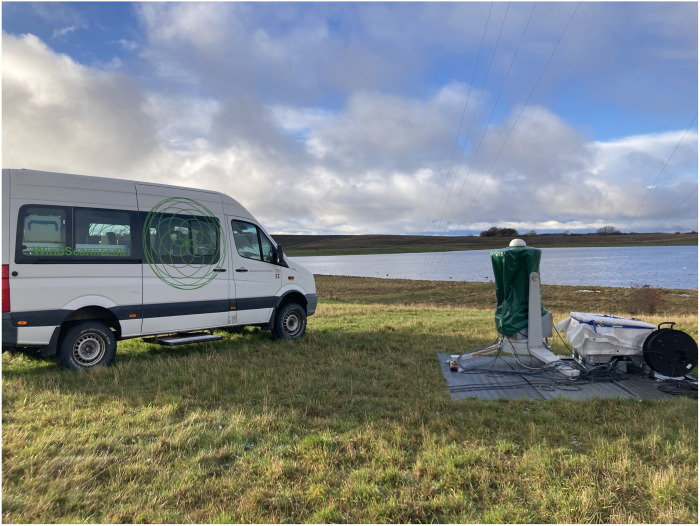
Short-range WindScanner. Photograph of a short-range WindScanner developed at the DTU and of the van used to both control the wind lidar and store the acquired data.

A WindScanner consists of a coherent Doppler wind lidar and an optical scanner (an example of such an instrument deployed in the field is shown in Fig. 6). The Doppler wind lidar uses a cw laser that emits an infrared radiation with a wavelength of 1.55 μm, which is transmitted into the atmosphere, and, subsequently, using a monostatic transceiver, the radiation backscattered by aerosols is collected through the same optical telescope.

The transmitted radiation is backscattered by particles (e.g., aerosols) that are suspended in the atmosphere and frequency shifted because of the Doppler effect. The received backscattered electromagnetic radiation from the measurement location is guided using an all-fiber architecture to a coherent homodyne in phase-quadrature detection scheme (*45*). This enables (i) the down-conversion of the frequency of the backscattered signal to frequencies that can be detectable by the electronics of a photodiode detector (i.e., approximately megahertz) and (ii) the detection of the sign of the frequency shift. Subsequently, the Doppler shift is estimated using the Fourier transform of the combined transmitted and backscattered signal. In particular, first, the power spectral density of the discrete Fourier transforms of the detected signals is calculated and block averaged to reduce the noise floor variance resulting in an effective sampling rate of about 322 Hz. A frequency estimator is lastly applied to estimate the wind-induced Doppler shift. A Doppler lidar thus measures the radial speed of the wind, which is the projection of the wind vector on the line of sight of the transmitted electromagnetic radiation.

**Fig. 7.**
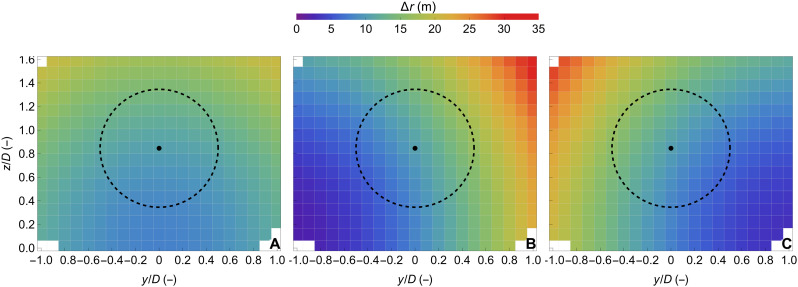
Spatial resolution of the three scanning wind lidars. The probe length of the line of sight per grid cell for each WindScanner corresponding to twice the Rayleigh length for each of the three wind lidars: (**A**) WS1, (**B**) WS2, and (**C**) WS3. The dashed circle marks the rotor diameter of 52 m, centered at the hub height of 44 m.

In the case of the cw wind lidars, the measuring distance is set optically by focusing the radiation via a diffraction-limited doublet lens. Measurement range adjustment is achieved by changing the distance between the fiber tip, where the radiation emanates, and the doublet lens. The drawback of this configuration is that the probe length, over which a radial speed is measured, scales with the distance to the power of 2, which means that the spatial resolution is decreased as the measuring distance increases. Therefore, the use of the WindScanner presented in this study is preferred at short ranges, typically <200 m, and the instruments are referred to as short-range WindScanners. On the basis of the setup used in this study, the probe length along the line of sight of each wind lidar, defined as twice the Rayleigh length of a focused Gaussian laser beam, is presented in Fig. 7. The probe length is found to vary between 9.2 and 23.7 m, 2.3 and 34.8 m, and 3.7 and 33.0 m, for the case of WS1, WS2, and WS3, respectively. Because of the location of the WS1 relative to the measuring plane (see Fig. 1B), the distribution of the probe length values of WS1 is approximately symmetric around the *z* axis. On the contrary, WS2 and WS3 have similar but mirrored characteristics regarding the distribution of the probe length values. The direction of the line of sight is steered using two individually controllable, rotating optical prisms that allow a high acceleration of rotation, which, combined with the high sampling rate, enables rapid scanning of a large volume of a flow field (*46*).

### Wind vector estimation

**Fig. 8.**
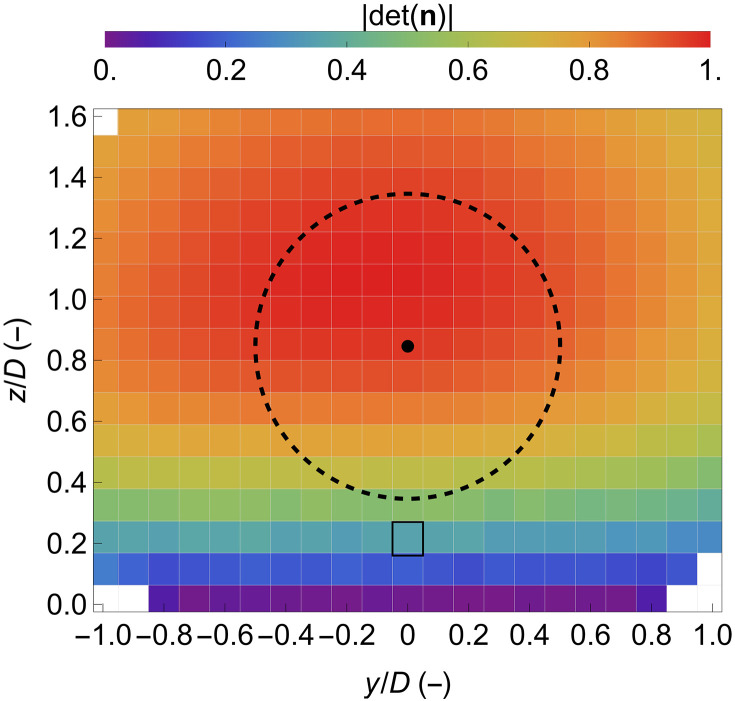
Determinant of the matrix used for the wind vector reconstruction. Contour plot of the determinant of the unit vector matrix n , which is formed by the line of sight of the three short-range WindScanners in each measurement location across the measured vertical plane. The black dot highlights the position of the center of the grid cells used to group the data. The dashed circle marks the rotor diameter of 52 m, centered at the hub height of 44 m, and the grid cell whose perimeter is highlighted by black denotes the location of the sonic anemometer installed on the downwind mast (see Fig. 1).

**Fig. 9.**
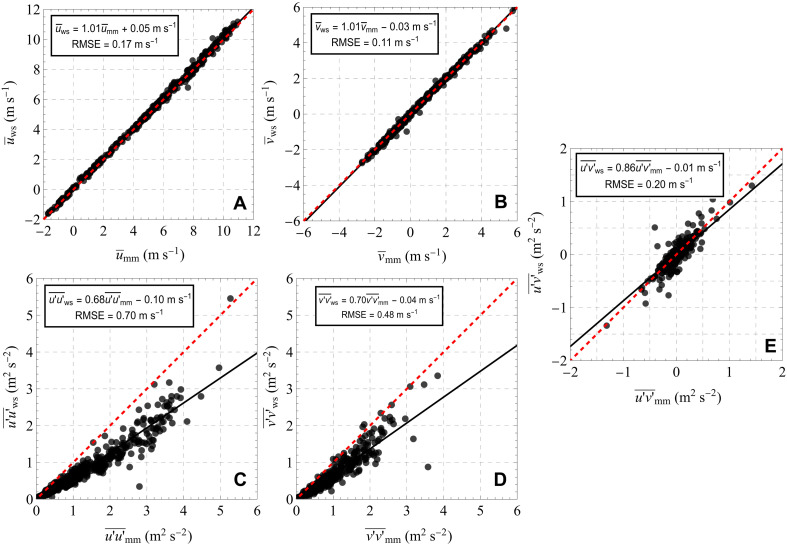
Comparison of wind statistics between the WindScanner wind lidars and a reference sonic anemometer. Scatter plots of the mean (**A** and **B**), the variance (**C** and **D**), and covariance (**E**) of the horizontal wind vector components at 10 m above the ground level, as measured by the WindScanners (statistics denoted by the subscript ws) and the sonic anemometer (statistics denoted by the subscript mm) installed at the downwind mast. The dashed red line indicates the 1:1 line, and the dashed black line is a Deming regression (the results are shown in the framed boxes in each scatter plot along with the corresponding root mean square error).

Three independent radial speed measurements are required to calculate the 3D wind vector at a given location (*26*, *27*). Let $n=\left\{\;n_{x},n_{y},n_{z}\;\right\}$ be the unit vector of a WindScanner wind lidar line of sight, then the radial speed $v_{r}$ at a distance r from the lidar is equal to the inner product of the unit vector n and the wind vector U(r) , i.e., $v_{r}\left(r\right)=n\cdot U\left(r\right)$ . The wind vector thus can be estimated by first inverting the unit vector matrix formed by the line of sight of the three wind lidars and subsequently multiplying the inverted matrix by the radial speed matrix. Figure 8 presents the determinant of the unit vector matrix n , which is formed by the line of sight of the three short-range WindScanners in each measuring location across the measured vertical plane according to the measuring configuration used in this study. We see that in the lowest heights, z<0.2D , the determinant is almost smaller than 0.2. Thus, at these low scanning heights, estimating the three wind vector components is impossible. In Fig. 9, we present the comparison of the mean, the variance, and the covariance of the horizontal vector components of the wind as measured by the WindScanners, 10 m above ground at a position where the sonic anemometer (USA-1 Sonic 3D, METEK GmbH, Germany) on the downwind mast was located (see Fig. 1C). The grid cell corresponding to the location of the sonic anemometer in Fig. 8 is highlighted in black. At that height, it is not possible to calculate statistics that include the vertical component of the wind vector; therefore, we focus the comparison only on the horizontal wind vector. This analysis is based on 421 half-hour periods that span the entire duration of the field campaign. Those periods were selected on the basis of having at least 50% data availability after the filtering processes of the wind lidar data, presented in the “Data selection and postprocessing” section, were applied. We observe a high correlation with biases of less than 0.1 m s^−1^ and root mean square error lower than 0.2 m s^−1^, in the mean longitudinal and transverse wind components. In the case of the variances and covariances, we see a very low bias, indicated by the values of less than 0.1 m^2^ s^−2^ in the offset of a regression analysis between the WindScanner and the sonic anemometer. However, we find that the turbulent fluctuations of both the longitudinal $\bar{u^{\prime }u^{\prime }}$ and the transverse $\bar{v^{\prime }v^{\prime }}$ components are underestimated by the WindScanner by ~30% due to the filtering induced by the wind lidars’ probe length. We observe a better agreement in the covariance of the longitudinal and transverse components $\bar{u^{\prime }v^{\prime }}$ . This could indicate that the length scale of turbulence that characterizes these fluctuations is comparable to or larger than the probe length of the WindScanners at the location where the sonic anemometer at the downwind was located. In that case, the impact of the wind lidars’ spatial resolution on measuring turbulent fluctuations would not be that substantial.

### Atmospheric conditions

The inflow wind conditions to the wind turbine were first assessed using the sonic and cup anemometers installed on the upwind meteorological mast (see Fig. 1, B and C). Sonic anemometers (USA-1 Sonic 3D, METEK GmbH, Germany) were installed at five different heights (i.e., 18, 31, 44, 57, and 70 m). However, data from the sonic anemometer at 57 m were not available for the period of the campaign. Therefore, only data from the remaining four sonic anemometers were included in this study. The sonic anemometer data used to (i) reconstruct the vertical profile of the horizontal wind speed, (ii) estimate the wind direction and subsequently the yaw misalignment of the wind turbine, (iii) provide a reference for the atmospheric turbulence intensity (i.e., the ratio between the SD and the mean horizontal wind speed), and (iv) assess the atmospheric stratification during the period of the field campaign. The sonic anemometer data were sampled at 50 Hz and postprocessed to first compensate for the flow distortion induced by the frame that holds the ultrasonic transducers, following the methodology presented in (*47*). In addition to the sonic anemometers, cup anemometers (Risø P2546A, WindSensor, Denmark) were installed in the mast at the same heights as the sonic anemometers and used as a reference for the inflow mean horizontal wind speed.

**Fig. 10.**
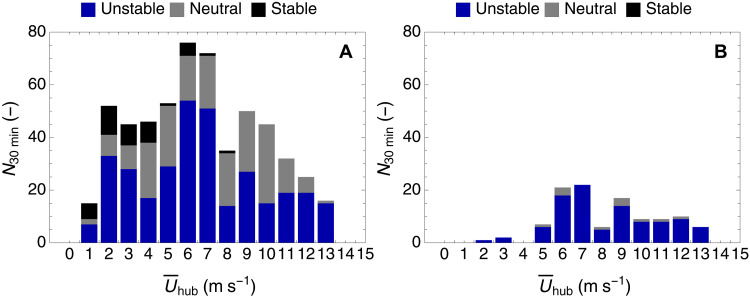
Atmospheric stability classification. Estimated atmospheric stability (**A**) for the whole period of the field campaign and (**B**) for the selected dataset used in the wake analysis based on the measurements of the sonic anemometer at the hub height (i.e., 44 m).

An important parameter that characterizes the wind conditions in the atmospheric boundary layer is the atmospheric stratification. For the assessment of the atmospheric stability, the sonic data were first rotated so that the mean longitudinal wind component $\bar{u}$ was aligned to the 30-min mean wind vector so $\bar{v}$ = $\bar{w}$ = 0. Subsequently, using the longitudinal cross-correlation of the wind vector component, we computed the friction velocity $u_{⋆}=\sqrt[{4}]{{\bar{u^{\prime }w^{\prime }}}^{2}+{\bar{v^{\prime }w^{\prime }}}^{2}}$ at each of the four measurement heights. In the final step, we estimated the Obukhov length scale (*48*)

$$L=-\frac{T}{\mathrm{\kappa g}}\frac{u_{⋆}^{3}}{\bar{w^{\prime }T^{\prime }}}$$ (1)

where $\bar{w^{\prime }T^{\prime }}$ is the vertical heat flux, T is the ambient air temperature (here, we use the temperature measurements provided by the sonic anemometers), κ=0.4 is the nondimensional von Kármán constant, and *g* = 9.81584 m s^−2^ the gravitational acceleration of Earth. Using the Obukhov length scale estimated at each height where a sonic anemometer was located, we estimated a height normalized stability parameter z/L , as an index of the atmospheric stratification. To characterize the atmospheric stratification we defined the following ranges: unstable stratification, z/L<−0.1 ; neutral stratification, ∣z/L∣≤0.1 ; and stable stratification, z/L>0.1. Figure 10A presents the results of the atmospheric stability characterization at hub height for different mean wind speeds. We find that most of the wind conditions during the period of the field campaign were characterized by unstable atmospheric stability.

### Data selection and postprocessing

#### Periodization

The lidar and mast data acquired during the period of the field campaign were segregated into consecutive 30-min periods.

#### Data selection criteria

Subsequently, the following criteria were used to select which periods should be investigated further:

1) Stratification homogeneity: The first criterion that we applied was that the atmospheric stratification estimation should be identical at all heights. This criterion eliminated cases where the blades of the wind turbine rotor could interact with layers of different atmospheric turbulence levels, as they crossed different height ranges while rotating. On the basis of this criterion, 28% of the 30-min periods were discarded. The remaining cases were 63% unstable, 31% neutral, and 6% stable.

2) Wind direction alignment: Subsequently, we aligned the coordinate system used in the sonic anemometers with the one used by the short-range WindScanner. Using the 30-min mean $\bar{u}$ and $\bar{v}$ components, we calculated the mean wind direction. Subsequently, we selected only those 30-min periods where the absolute difference between the wind direction and the coordinate system of the short-range WindScanner was equal to or less than 7.5°. This criterion was applied to avoid examining cases where the wake was wholly or partly outside the vertical plane measured by the short-range WindScanner. After the filtering, a total of 110 half-hour periods were selected with 99 corresponding to unstable atmospheric stability and 11 corresponding to neutral atmospheric stability (see Fig. 10B).

3) Mean wind speed: The final step was to split the data based on the 30-min mean wind speed at hub height (i.e., 44 m) using the sonic anemometer data. A histogram of the mean wind speeds is shown in (see Fig. 10B), where we see that the most common mean wind speed at hub height was equal to 7 m s^−1^.

#### The final selection

**Fig. 11.**
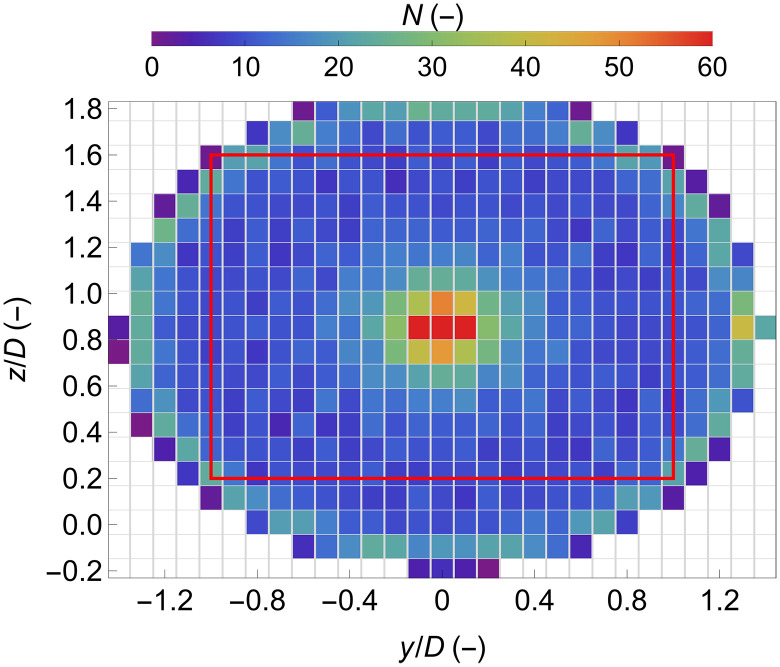
Number of measurements per grid cell per WindScanner. The red rectangle highlights the area, which was used to study the wake characteristics.

In the present analysis, we used as a case study the 22 half-hour mean periods during which the mean wind speed at the hub height was equal to 7 m s^−1^. The selection of this wind speed bin was made because it contained the most data. The WindScanner data processing involved the following steps:

1) Block averaging: The Doppler spectra were block averaged to decrease the resolution of the time series from 322 to ~100 Hz to reduce the noise variance across the frequency bandwidth of the Doppler spectra.

2) Noise normalization: The averaged Doppler spectra were normalized by dividing with a background noise spectrum.

3) Spatial binning: The normalized Doppler spectra were gathered in grid cells according to the coordinates of each measurement. Figure 11 presents the number of measurements per grid cell per WindScanner for one scan. We can see that because of the scanning pattern used (presented in Fig. 2), more measurements are acquired at the center of the scanning plane in comparison to the periphery. Overall, in the area used to investigate the wake characteristics (enclosed by a red rectangle in Fig. 11), at least 10 measurements (sampled at ~100 Hz) were available per grid cell.

4) Spectral area normalization: Each Doppler spectrum in a bin was normalized with the area of the Doppler peak.

5) Spatial averaging: The spatially binned Doppler spectra were averaged by taking into account both the grid cell location of the measurements and the time that the trajectory is passing through a grid cell.

6) Radial speed estimation: The radial wind speed of each averaged Doppler spectrum was calculated using a Doppler shift estimator.

7) Outlier removal: The data were gathered per grid cell and per 30-min period, and radial wind speeds that were found below or above the lower and higher outer fences were filtered out.

8) Wind statistics: The mean, the variance, and the covariance of the reconstructed wind vector in each grid cell were estimated.

### Wake center

To estimate the wake statistics, we first calculated the center of the wake in each 30-min period. Subsequently, the statistics over the ensemble for each wind speed bin were used in a moving frame of reference based on the location of the wake center. The determination of the wake center was performed on the basis of the following steps:

1) Inflow normalization: The longitudinal wind speed estimation in each grid cell was normalized by dividing it by the corresponding value of the free vertical profile at the height of the grid cell. The estimation of the vertical profile was performed using the mean vertical profiles measured at *y* > 50 m and *y* < −50 m. For the wind direction sector selected and from the observed spread of the wind speed deficit in the wake, it is expected that at the transverse distances ∣y∣>50 m, the wind speed measurements per height are not distorted by the wake of the wind turbine.

2) Wake center determination: The center of the wake for each 30-min period was found from the center of a 2D Gaussian model fitted to the data. In the model, we allow the spread of the wake to be different in the *y* and *z* axes.

**Fig. 12.**
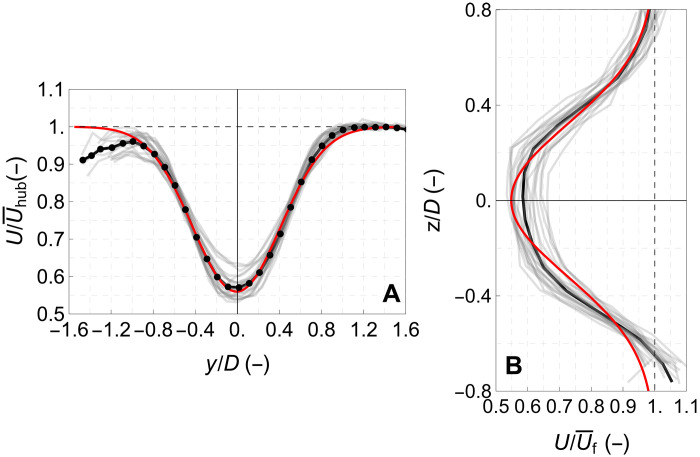
Wake profiles. (**A**) Horizontal and (**B**) vertical profiles of the velocity deficit around the center of the wake. Gray lines depict the profiles of each of the individual 22 half-hour periods examined, black line is the corresponding mean, and red line is a Gaussian fit.

In Fig. 12, we present the horizontal and vertical profile of the velocity deficit around the center of the wake. The velocity has been normalized either by the hub height speed ${\bar{U}}_{\text{hub}}$ (in the case of the horizontal profile in Fig. 12A) or by the free wind profile ${\bar{U}}_{f}$ (in the case of the horizontal profile in Fig. 12B). We observe that especially in the case of the wake’s horizontal profile, the velocity deficit has a trend that can be approximated by a Gaussian distribution (depicted with the red curve in both the plots in Fig. 12). This trend is in agreement with numerical predictions of the wake characteristics of this wind turbine generated using a computational fluid dynamics model for a downwind distance approximately the same as the one investigated in this study (*49*).
