# Supplementary material for: Experimental characterization of complex atmospheric flows: A wind turbine wake case study
Source: Sci Adv. 2025 Nov 21;11(47):eadw8524. doi: 10.1126/sciadv.adw8524 (PMC12637304; doi:10.1126/sciadv.adw8524)
Supplement: Supplementary file 1 — Legends for supplementary data files [file sciadv.adw8524_sm.pdf]

Science Advances
AAAS

Supplementary Materials for

Experimental characterization of complex atmospheric flows: A wind turbine wake case study

Nikolas Angelou *et al.*

Corresponding author: Nikolas Angelou, nang@dtu.dk

Sci. Adv. **11**, eadw8524 (2025)
DOI: 10.1126/sciadv.adw8524

The PDF file includes:

Legends for supplementary data files

Other Supplementary Material for this manuscript includes the following:

Supplementary data files

Supplementary Text

The zipped file *SupplementaryFile_Data.zip* contains 8 data files with a comma separated values format. These data files have been used for i. the comparison between the estimated wind statistics using the WindScanner wind lidars and the meteorological masts (i.e Figs. 3 and 9) and ii. for the study of the wake characteristics (i.e Figs. 4, 5 and 12). The label of the each file corresponds to the number of the figure in the manuscript. A header that denotes the parameters and the corresponding units prepends the data in each file.
